# How Does Entrepreneurial Self-Efficacy Influence Innovation Behavior? Exploring the Mechanism of Job Satisfaction and Zhongyong Thinking

**DOI:** 10.3389/fpsyg.2020.00708

**Published:** 2020-05-08

**Authors:** Jiangru Wei, Yuting Chen, Yamin Zhang, Jing Zhang

**Affiliations:** School of Management, Nanjing University of Posts and Telecommunications, Nanjing, China

**Keywords:** entrepreneurial self-efficacy, job satisfaction, innovation behavior, Zhongyong thinking, entrepreneurial education

## Abstract

Innovation behavior for entrepreneurship is known as a driving force to obtain competitive advantages. As a key quality for entrepreneurial success, the mechanism of entrepreneurial self-efficacy (ESE) acting on innovation behavior needs further verification, which has led to the primary objective of this paper via applying the Goal Self-Concordance Theory, as well as to further building a theoretical model. Two hundred forty-nine samples of Chinese entrepreneurs have been empirically analyzed in this study, contributing to the following findings. Firstly, ESE has significantly positive effects on entrepreneurial innovation behavior. Secondly, job satisfaction plays a mediating role between ESE and innovation behavior. Thirdly, Zhongyong thinking moderates the relationship between ESE and job satisfaction. The research results might deliver great value in cultivating ESE, encouraging positive entrepreneurial attitude, enhancing job satisfaction, and ultimately inspiring innovation behaviors.

## Introduction

The essence of entrepreneurship is to seize opportunities, integrate resources, and then innovate and act promptly (Johnson, 2001; Stephens et al., 2013). Innovation behavior (IB) for entrepreneurship is not only the key to cope with dynamic changes in external environment for survival but also the driving force to further obtain competitive advantages (Amo, 2010; Huarng and Ribeiro, 2014). Thus, the promotion of IB has become an important goal in entrepreneurship (Erikson, 2002), while the approach has yet been partly explained by social capital and institutional impact (Chen and Zhou, 2017; Stephens et al., 2013). Instead, more factors should be counted to analyze the IB mechanism, especially the personal and cultural characteristics of entrepreneurs (Robson and Obeng, 2009; Nathan and Lee, 2013; Barakat et al., 2014).

Personal characteristics of entrepreneurs are considered as playing critical roles in environmental adaptation and personal achievement (Byrne and Shepherd, 2013), such as perceived discrimination in immigrant entrepreneurship (Robertson and Grant, 2016) and self-cognition in entrepreneurial career decision making (Obschonka and Stuetzer, 2017). Self-efficacy refers to the belief of whether one person can achieve certain goals (Gist, 1987; Fast et al., 2014), and the Social Cognitive Theory regards self-efficacy as an important determinant of behavior (Bandura, 1991). Entrepreneurial self-efficacy (ESE) is the application of self-efficacy in entrepreneurship research, referring to the extent to which entrepreneurs are confident about their own entrepreneurial skills to complete various tasks and projects (Boyd and Vozikis, 1994; Chen et al., 1998). The process of entrepreneurship is full of setbacks, which requires entrepreneurs with good psychological qualities. ESE, a typical characteristic of entrepreneurs, represents the belief and attitude of entrepreneurs to overcome various difficulties and achieve entrepreneurial success (Gist and Mitchell, 1992; Chen et al., 1998). Existing studies have proved that ESE contributes greatly to the prediction of entrepreneurial intention and promotion of entrepreneurial performance (Hmieleski and Corbett, 2008; Caines et al., 2019). Bandura (1991) proposed that personal choice, effort, and behavior are affected by self-cognition of their abilities, which suggests that ESE can also be a predictor of behaviors associated with IB (Barakat et al., 2014). Recent research has shown that entrepreneurs with higher ESE are more likely to set innovation-related goals for their companies and more willing to exhibit innovative behavior (Drnovsek and Glas, 2008; Chen and Zhou, 2017). Nevertheless, few entrepreneurs really translate their ESE into IB in practice, even though many people are confident in the performance of innovation and the achievement of entrepreneurial goals before action (Markman and Baron, 2003). Most entrepreneurs have to quit after suffering various entrepreneurial risks and challenges, as well as psychological pressure and emotional exhaustion (Bradley and Roberts, 2004; Kasouf et al., 2015). However, previous studies mostly focused on the direct impacts of ESE on IB (Ahlin et al., 2013; Barakat et al., 2014; Chen and Zhou, 2017). We still have limited understanding of the process of how ESE influences IB and even less empirical evidence on the mechanism research. Thus, it is valuable to study the process of ESE influencing IB for entrepreneurial practitioners to carry out innovation-related activities effectively.

Cultural characteristics rooted in historical development have a profound and everlasting impact on the way of thinking and behavior of individuals and groups (Cardon et al., 2011), which reveals the cultural aspect of entrepreneurial activities (Nathan and Lee, 2013). Empirical studies have shown that cultural characteristics in entrepreneurial activities have a positive impact on the creation of regional wealth (Fritsch and Wyrwich, 2016; Bacq and Eddleston, 2018). Cultural characteristics of innovation activities are increasingly prominent (Morris and Allen, 1994; Wry et al., 2011), appearing to be a dominant factor to understand the way of thinking and behavior of entrepreneurs concerning a specific cultural background, as well as to effectively carry out global entrepreneurship cooperation (Amo, 2010). Although the cultural characteristics are embodied in entrepreneurial activities (Mcgee et al., 2009), the understanding of entrepreneurs’ cognition and behavior from the cultural perspective is still insufficient. To fill these research gaps, studies are required on the process mechanism of how ESE influences IB under certain cultural boundary conditions to ensure successful completion of entrepreneurial activities.

The Goal Self-Concordance Theory provides a general theoretical framework through which ESE can influence IB. This theory asserts that people will have more subjective well-being and a higher level of goal accomplishment when goals are driven by their authentic choices rather than a sense of control by external forces (Sheldon and Elliot, 1999; Sheldon and Marko, 2001; Judge et al., 2005). Self-concordant goals represent people’s actual interests and passions as well as their central values and beliefs. People are willing to make sustained efforts and feel more satisfied with self-concordant goals, which are more likely to be achieved (Downes et al., 2016). Moreover, Sheldon et al. (2004) suggested that the effects of self-concordance may be moderated by culture factors, which are considered as having a deep-rooted impact on cognition and behavior (Nathan and Lee, 2013). Although self-consistency generally exerts positive effects on subjective well-being regardless of cultural differences, cultural factors do influence individual perception of satisfaction and the degree of achievement of self-concordant goals, given likely cultural differences in the strength of social pressures and expectations (Sheldon et al., 2004).

IB is often regarded as an important activity and goal by entrepreneurs (Erikson, 2002) because promotion of IB is consistent with an individual’s interest in entrepreneurship and self-development (Huarng and Ribeiro, 2014). In other words, promoting IB is a self-concordant goal of entrepreneurs. According to the Goal Self-Concordance Theory (Sheldon and Elliot, 1999), individuals are more willing to make repeating efforts and to embrace changes in well-being to achieve self-concordant goals, which implies that job satisfaction (JS) correlates with well-being, which affects IB (Gallivan, 2003; Xerri, 2014). At an entrepreneurial workplace, JS, a comprehensive evaluation of the feelings about one’s work, describes the cognition of working status (Wright and Cropanzano, 2000). Studies have shown a positive correlation between ESE and JS (Bradley and Roberts, 2004; Baldwin et al., 2006), for entrepreneurs with high ESE gradually set expectations for work and enhance JS in the process of realizing self-concordant goals (Tolli and Schmidt, 2008). Furthermore, corresponding changes in satisfaction are considered positively correlated with IB (Trivellas and Santouridis, 2009), which implies that JS may have a supportive effect to convert personal characteristic advantages (ESE) into effective action (IB). Therefore, this paper perceives JS as a significant mediator for the study of ESE and IB.

Cultural capital is perceived as having a profound and permanent impact on the way of thinking and behavior in entrepreneurial activities (Nathan and Lee, 2013). In this study, evidence has been derived from Chinese entrepreneurs through investigating how ESE can effectively influence their IB and enable them as one of the most active innovation groups in global entrepreneurship (Liu et al., 2015). Zhongyong (ZY) thinking, a representative cultural capital in Chinese Confucianism, is characterized as a thinking mode of how majority of Chinese view things, people, and environment (Chou et al., 2014). Individuals with ZY tend to reflect from multiple perspectives and achieve goals in harmoniousness with a holistic viewpoint (Wu and Lin, 2005). Varying from western countries, ZY has a profound impact on the way of thinking and behavior of Chinese entrepreneurs and thus can be referred to as a boundary condition for learning entrepreneurs’ attitude and behavior. This paper proposes that ZY is a moderating variable of the relationship between ESE and JS.

Therefore, this paper is meant to explore the influencing mechanism between ESE and IB based on the Goal Self-Concordance Theory and to further verify the mechanism via empirical research involving 249 participants from China. This paper inspires future relevant researchers and practitioners by shedding light on the following findings. To start with, this paper provides more empirical evidence from Chinese entrepreneurs that ESE is positively correlated with IB, indicating that an entrepreneur’s psychological capital plays an active role in his/her behavior (Mcgee and Peterson, 2017). Furthermore, this study has found that JS mediates the influence of ESE on IB, contributing to a more comprehensive understanding of the influencing mechanism of ESE and IB. This study applies JS to entrepreneurial management and increases the general cognition that positive attitudes or feelings can promote beneficial behavior (Griffin et al., 2001). Finally, this paper discusses the cultural boundary effect of ZY thinking on the relationship between ESE and JS from a perspective of the Chinese indigenous construct, enabling better apprehension of the innovation activities of Asian entrepreneurs and possibility of carrying out cross-cultural innovation activities (Knight, 1987).

## Theory and Hypotheses

### Entrepreneurial Self-Efficacy and Innovation Behavior

In Bandura’s research, ESE appeared as a new concept applied in the field of entrepreneurship in the 1990s and was regarded as a relatively stable psychological capital of entrepreneurs (Ibrayeva, 2006). ESE refers to the self-confidence intensity of entrepreneurs on whether their own entrepreneurial skills can complete various entrepreneurial activities, reflecting the belief that entrepreneurs are equipped with the competency to influence their surroundings and succeed through corresponding actions (Boyd and Vozikis, 1994; Chen et al., 1998). As a kind of belief in accomplishing a certain goal or task, the concept of ESE has been accepted as useful to explain the development of entrepreneurial intention and the decision-making process afterward (Liu et al., 2019). Influenced by environmental and personal factors, entrepreneurs are able to reinforce their ability to cope with negative emotions and pressures while continuously exposed to an entrepreneurial environment (Gist and Mitchell, 1992; Shepherd, 2004), so their ESE can be obtained, modified, and enhanced (Chen et al., 1998; Barz et al., 2015), and to further affect their performance via utilizing originality, resourcefulness, and other skills (Gist and Mitchell, 1992; Mcgee and Peterson, 2017).

Starting from the aspect of process flow, Scott and Bruce (1994) pointed out that to conduct IB, individuals have to seek support for their ideas and establish alliances to realize the ideas through the buildup of prototypes or models, and finally lead to new products or services. Innovation is a complex process along the generation, promotion, and practice of new ideas (Brown and Duguid, 1991; Kazadi et al., 2016), and IB is regarded as the behavior by which individuals generate new ideas or solutions after identifying and analyzing problems, and further support-seeking, capacity recognition, and practice (Scott and Bruce, 1994; Kang et al., 2016). In the field of entrepreneurship, IB can be demonstrated in different stages of planning, organizing, implementing, and controlling (Beaver and Prince, 2002). IB is closely related to entrepreneurial creativity, which is promoted and constrained by many mechanisms, including perception, motivation, knowledge, ability, and belief (Barakat et al., 2014). On the other hand, ESE has been proved to correlate with several behaviors, such as opportunity identification and failure learning, as well as innovation associated with entrepreneurship (Chen et al., 1998; Dempsey and Jennings, 2014).

According to the Goal Self-Concordance Theory (Sheldon and Elliot, 1999), those who pursue self-concordant goals will make continuous efforts to achieve their goals and are more likely to succeed. That is, self-concordance has a positive effect on achieving goals under the circumstance that individuals have full self-consciousness. Previous self-concordance research has recognized goal-specific efficacy as a variable closely related to autonomous motivation and as one of the important antecedents to goal accomplishment (Judge et al., 2005; Downes et al., 2016). IB is frequently recognized as an important activity and goal by entrepreneurs (Erikson, 2002). As a psychological self-cognition of entrepreneurs, ESE may affect their IB in different ways as below. Firstly, the entrepreneurial environment is full of opportunities, and innovation performance in entrepreneurship can be associated with psychological satisfaction by entrepreneurs with high ESE (Chen et al., 1998). Secondly, innovation is a process characterized by risks and uncertainties, and people with high ESE are more capable of embracing the reality (Mcgee and Peterson, 2017). To follow that, people with ESE set higher expectations on results than those at a lower ESE level, who prefer to be conservative while setting innovation goals and practice (Tolli and Schmidt, 2008; Caines et al., 2019). In a word, entrepreneurs with a great sense of ESE are more confident in achieving self-concordant goals and more likely to overcome difficulties in the process of innovation, which stimulates the modification and reinforcement of ESE as a return. On the contrary, individuals with low ESE often doubt their ability of innovation; hence, they are prone to avoiding problems or even quitting when encountering obstacles, especially when they are emotionally exhausted (Neumeyer et al., 2018). Based on the above theoretical analysis and deduction, this paper has reached the following hypothesis:

H1: Entrepreneurial self-efficacy has a positive effect on entrepreneurs’ innovation behavior.

### Entrepreneurial Self-Efficacy and Job Satisfaction

Job satisfaction (JS) refers to the feelings that employees observe to evaluate their work or work experiences concerning previous experiences, current expectations, or available alternatives (Perdue et al., 2007). The construction of JS is a complex, multidimensional, and interrelated entity of tasks, roles, relationships, and rewards (Cranny et al., 1992). JS usually consists of five interrelated subordinate elements, including satisfaction about tasks assigned, salary, promotion, supervisor, and co-workers (Tsui et al., 1992).

The Goal Self-Concordance Theory supports the view that individuals are more willing to complete goals that comply with their own intentions and interests, which contribute to a broad subjective well-being (Sheldon and Elliot, 1999). Furthermore, Judge et al. (2005) developed the theory to link self-consistency with JS, suggesting that self-efficacy, as an important component of core self-evaluations, can improve JS. Entrepreneurship is often the embodiment of strong entrepreneurial intention of entrepreneurs, who are eager to put creative ideas into reality, and ESE is regarded as the dominant drive to transform potential entrepreneurs into nascent entrepreneurs (Erikson, 2002). Self-concordant goals motivate entrepreneurs to try harder and be empowered to handle challenges (Hwang et al., 2016), since entrepreneurs with high self-efficacy gradually attain work expectations and ideal psychological states, which are closely related to the enhancement of JS (Baldwin et al., 2006; Jean and Mathieu, 2015). In addition, individuals with high ESE are more competent to act in the role of entrepreneur, as well as to collect, integrate, and make use of relevant entrepreneurial information (Barakat et al., 2014). Interestingly, ESE, including factors related to emotions and skills, can be strengthened after constant interaction with the entrepreneurial environment (Barz et al., 2015), which seems to form a virtuous circle. One of the benefits of improving ESE is that entrepreneurs can better deal with interpersonal relationships in new ventures, which are often considered as an important factor of JS (Cranny et al., 1992). In a word, entrepreneurs with a high ESE can carry out effective environmental recognition, psychological cognition, and interpersonal interaction, which contribute to a higher level of JS. The above analysis has enabled the following hypothesis:

H2: Entrepreneurial self-efficacy has a positive effect on job satisfaction.

### The Mediation Effect of Job Satisfaction

The happy-productive worker hypothesis has most often been examined in organizational research (Wright and Cropanzano, 2000), among which the positive impact of JS on performance has been widely considered (Lee and Chang, 2008). However, there is a lack of discussion on the entrepreneurial process, especially the cognitive process of entrepreneurs, for the public concentrate more on their performance (Boso et al., 2013). Previous self-concordance research has pointed out that enhanced JS may conduce to more positive goal attainment (Sheldon and Marko, 2001), and entrepreneurs are inclined to make endeavors to realize self-concordant goals and improved well-being, which will further affect the behavior of entrepreneurs (Trivellas and Santouridis, 2009).

As a positive psychological experience in the process of entrepreneurship, JS is also known to shape work behavior in entrepreneurial research (Gallivan, 2003; Niu, 2014). Previous studies have been done with proof that JS is associated with certain positive behaviors and outcomes, such as lower emotional stress (Hayes et al., 2013), better organizational citizenship behavior (Li et al., 2010), work performance (Bond and Bunce, 2003), and innovation performance (Chen et al., 2012). It has been recently claimed that JS impacts IB in a positive way (Niu, 2014). There is more empirical evidence showing that entrepreneurs with higher JS, as well as higher self-confidence, are more self-motived to interact with their surroundings and promote innovation behaviors through information and idea exchange (Xerri, 2014). Innovation is a process with inevitable risks (Kang et al., 2016). Higher JS indicates that it is easier for entrepreneurs to deal with changes of environment and interpersonal conflicts, which results in less pressure and emotional exhaustion (Li et al., 2010), so as to inspire and sustain innovation behaviors (Wu et al., 2011). As discussed above, entrepreneurs with higher ESE have more advantages in environmental recognition, psychological cognition, and interpersonal interaction, which are beneficial to improve JS. Thus, higher ESE is considered in this paper to be able to improve JS and further positively affect IB. Therefore, this has led to the third hypothesis in this paper:

H3: Job satisfaction mediates the relationship between entrepreneurial self-efficacy and innovation behavior.

### Moderating Effect of Zhongyong Thinking

Entrepreneurs’ thinking and behavior are inevitably influenced by cultural factors. ZY thinking (the Doctrine of the Mean) originated from Confucian philosophy and was developed by ancient Chinese scholars, making ZY one of the core thinking modes of Chinese people for thousands of years (Chang and Yang, 2014). Chinese people are more likely to avoid an extreme perspective when confronting contradictions and conflicts, hence having a moderate way to respond, as well as when making decisions and taking actions (Peng and Nisbett, 1999; Lee, 2000). As a Chinese indigenous construct that reflects the thinking of Confucian heritage cultures (Pan and Sun, 2017), ZY is complicated cognitive thinking about how Chinese think about objects, people, and environment (Hong, 1978; Pierce and Aguinis, 2013; Chou et al., 2014). The connotation of dialectical thinking is similar to ZY thinking (Peng and Nisbett, 1999), while ZY is more composed of holistic thinking and changing (Zhou et al., 2019). The primary qualitative research on ZY in psychology started in the 1990s (Yang and Zhao, 1997). Yang (2009) regarded ZY as a unique metacognitive-level practical thinking system, involving skills of planning, monitoring, and evaluating progress during task completion (Yang and Lin, 2012; Qu et al., 2018). Specifically, ZY refers to a thinking mode about how to integrate both external conditions and internal needs from multi-perspectives and to take practical actions in a specific situation (Wu and Lin, 2005). The core values of ZY are eclectic and integrated thinking (Yang and Zhao, 1997; Zhou et al., 2019). Wu and Lin (2005) proposed three features to materialize the doctrine, including multi-thinking, integration, and harmoniousness (Chou et al., 2014; Chang and Yang, 2014; Pan and Sun, 2017). Among them, multi-thinking is a way of thinking by which individuals recognize the dialectical relationship between contradictory elements and achieve dynamic balance through a mutually complementing and promoting process (Pan and Sun, 2017). People with ZY tend to consider from multiple dimensions, in terms of time, space, and roles, which helps them make long-term plans and adjust to dynamic circumstances. Integration refers to how an individual should consider external conditions (e.g., different opinions from others and limited material resources) and internal needs to reach a consensus, indicating that ZY stresses the consolidation of both external circumstance and individual needs but also focuses on the connection between objects, people, and the environment from a holistic viewpoint (Chang and Yang, 2014). Thus, individuals with higher ZY can adjust their opinions in combination with external conditions and integrate their own viewpoints into the thinking of others. The harmoniousness is an ideal state for relationships and a means of dealing with conflict relationships so as to avoid extreme reactions (Qu et al., 2018). People with ZY target achieving goals harmoniously and making reasonable choices after taking internal and external conditions into account. Therefore, ZY indicates the idea of making progress with time and environment, not only as one of the Chinese cultural treasures but also as a cognitive strategy to effectively cope with changes and the uncertain environment nowadays.

**FIGURE 1:**
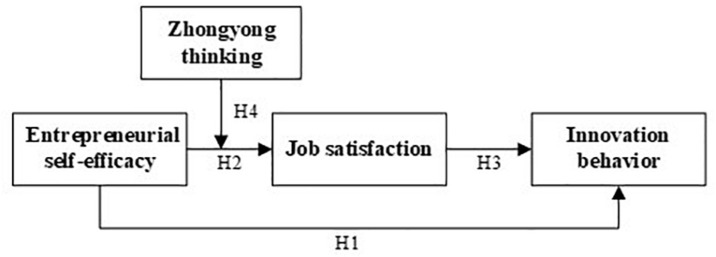
Conceptual model of entrepreneurial self-efficacy influencing innovation behavior.

With a large degree of autonomous function, goals are considered as unique cognitive structures in the Goal Self-Concordance Theory, and self-consistent goals represent personal interests and deep-rooted values (Sheldon and Elliot, 1999). The process from goal setting to realization is a necessary process for individuals to internalize national cultural values. Individual perception of satisfaction and the degree of achievement of self-concordant goals may be moderated by culture factors (Sheldon et al., 2004). For a long time, Chinese culture has regarded ZY thinking as one of the most important metacognitive factors regulating people’s emotions and attitudes (Yang, 2009). Although there is still a lack of cross-cultural studies in the existing literature on entrepreneurship, Chou et al. (2014) demonstrated that individuals with a higher level of ZY thinking are more capable of coping with work stress and transforming challenge-related stress into JS. As an important mode of cognitive thinking of Chinese entrepreneurs (Pierce and Aguinis, 2013), ZY thinking can also be an effective cognitive strategy that can give full play to the positive effect of ESE on JS. First of all, entrepreneurs with a greater attitude toward ZY prefer multi-thinking and would evaluate JS from a long-term perspective (Chou et al., 2014), implying that they are less likely to be misled by negative feelings. In addition, they are less affected by stress at work and negative emotions, given that multi-thinking helps to weaken contradiction and adapt to environment change in the process of entrepreneurship. Secondly, the connotation of integration in ZY motivates entrepreneurs to develop the ability to integrate a variety of resources, such as professional knowledge, human resources, and financial capital (Wu and Lin, 2005). In that case, they repeatedly think, learn, and then optimize, so that they can effectively and efficiently solve problems and eventually achieve goals, resulting in higher JS (Yang, 2009). Thirdly, ZY facilitates in accomplishing entrepreneurial goals via selecting feasible options in a combination of multiple factors (Zhang and Gu, 2015), such as cost efficiency and utility maximization. This represents a kind of self-consistency (harmoniousness), that is, entrepreneurs with a higher level of ZY can apply self-consistent methods to reach entrepreneurial ambitions, which are undoubtedly in accordance with the need for satisfaction of entrepreneurs and further lead to higher JS. In general, entrepreneurs with higher ZY thinking can cope with entrepreneurial pressure, integrate resources, and implement suitable methods, which obviously promotes the effective role of ESE and thus increases JS. As a result, this study has developed the following hypothesis, with a conceptual model shown in Figure 1.

H4: Zhongyong thinking positively moderates the relationship between entrepreneurial self-efficacy and job satisfaction.

## Materials and Methodology

### Participants and Procedure

Hebert and Link (1989) defined the entrepreneur as someone who specializes in taking responsibility and making judgment decisions of a company. Entrepreneurs are more inclined to take on a variety of responsibilities of start-up activities due to the shortage of human and environmental resources (Zhao et al., 2012). They are not only the decision makers but also usually the executors of the companies to ensure the effective implementation of start-up decisions. From the perspective of execution, top managers are crucial practitioners who can transform ESE into behavior, and their feedback based on practice can affect the revision and re-execution of decisions. Thus, we took founders and top managers as co–decision makers, and they are responsible for the business. Hmieleski and Corbett (2008) also took founders and top management team leaders as participants in an empirical study on ESE. Therefore, our participants were start-up founders and top managers, who are familiar with their company’s products and processes. Given the Chinese context, the policy factor is often an important factor to drive the implementation of entrepreneurship. Industries such as electronic information, internet work, and financial service are representative to study IB of entrepreneurs because of preferential policies offered by the Chinese government. One supportive policy is that physical space and infrastructure for newly established small and medium-sized technology-based enterprises can be provided by a business incubator, which has attracted many innovative entrepreneurs. Thus, we chose to conduct a questionnaire survey in a Chinese business incubator, where samples can be found intensively and conveniently.

We sent emails with instructions and questionnaires to start-up founders and top managers. In the email, as explained and emphasized in the survey, all participants could freely drop out in the process, ensuring it will be done anonymously, without involving any commercial interests. All participants read the participant information statement and provided online informed consent prior to the questionnaire. Two hundred ninety-two questionnaires were given feedback, and we eliminated 43 invalid questionnaires and finally obtained 249 valid questionnaires, with an effective rate of 85.27%. Participates were from eight provinces, including Jiangsu, Henan, Shanghai, Anhui, Xinjiang, Beijing, Hubei, and Fujian. The samples consisted of 140 males (56.22%) and 109 females (43.78%), primarily under the age of 40 (*n* = 219, 87.95%), and more than 80% of them had a bachelor’s degree or above (*n* = 220, 88.35%). Sixty-eight (27.31%) participants had less than 1 year experience as start-ups, 64 (25.70%) with 1–3 years, and 117 (46.99%) with more than 3 years of working experience. The composition of the samples is shown in Table 1.

**TABLE 1:** Sample description.

Individual characteristics	Category	Quantity	Percentage
Gender	Male	140	56.22%
	Female	109	43.78%
Age	≤25	68	27.31%
	26–30	88	35.34%
	31–35	45	18.07%
	36–40	18	7.23%
	≥ 41	30	12.05%
Education background	High school and below	12	4.82%
	Diploma	17	6.83%
	Bachelor	153	61.44%
	Master and above	67	26.91%
Experience in the start-up	≤1 year	68	27.31%
	1–5 years	64	25.70%
	≥5 years	117	46.99%

*The tail difference of percentages is adjusted at the end of each item; N = 249.*

### Measurement of Variables

In this study, ESE, JS, IB, and ZY were measured by five-point Likert scales, ranging from 1 (strongly disagree) to 5 (strongly agree); meanwhile control variables were converted to dummy variables. The survey instruments of ESE, JS, and IB were originally constructed in English but translated into Chinese. There has been version modification incorporating language differences to make sure of the accuracy in both English and Chinese contexts.

#### Entrepreneurial Self-Efficacy

ESE was measured by four question items adopted from the studies of Mcgee et al. (2009) and Barakat et al. (2014). Sample items include “I am able to choose suitable employees for my business,” “I am able to come up with new ideas to solve problems in entrepreneurship,” and “I have confidence in my ability to solve problems in my business.”

#### Job Satisfaction

There are many factors that might influence JS, concerning the type of industry, work, and working environment (Bhoganadam and Dasaraju, 2015). We adopted a six-item scale adapted from Tsui et al. (1992) for measurement purposes, including judgment on advancement, financial returns, and interpersonal relationships, which is applicable to measuring JS of entrepreneurs in different start-ups. For instance, “I am satisfied with the opportunities which exist in this organization for advancement,” “I am satisfied with the payment I receive for my job,” and “I am satisfied with the relations with others in the organization with whom I work.”

#### Innovation Behavior

IB was measured with the single dimensional scale introduced by Scott and Bruce (1994), which has been widely accepted and used with reliability and validity (Kang et al., 2016). The scale includes six items, such as “I always seek to apply new processes, techniques and methods”; “I often communicate with others and present my new ideas”; and “In order to implement new ideas, I can find ways to get the resources I need.”

#### Zhongyong Thinking

Multi-thinking, integration, and harmoniousness are the key characteristics of ZY thinking (Chou et al., 2014); thus, the three-dimensional scale proposed by Wu and Lin (2005) was applied to measure ZY. The multi-thinking dimension included four items, such as “I am used to thinking about one thing from different perspectives.” The integration dimension consisted of five items, such as “I often try to find acceptable opinions in a situation of disagreement.” The harmoniousness dimension includes four items, such as “I usually adjust my behavior for overall harmony.”

#### Control Variables

Apart from those, two demographic and two additional control variables were measured in this study. Previous studies have indicated that factors such as gender, age, education background, and experience in the start-up related to IB. Therefore, control on the factors mentioned above has been strictly followed while selecting participants. First, we control for the gender of the participants, as some research has indicated that innovation performance differs between males and females (Kang et al., 2016). Second, the age of the participants is controlled because research has indicated that differences in innovation propensity can be partly explained by gender and age (Rizzuto, 2011). Third, education background is considered to affect individuals’ cognitive abilities and stock of knowledge, which are correlated with IB (Wu et al., 2011). We also controlled measures of experience in the start-up, as studies have shown that related human and social capital influence innovation effectively (Ng and Feldman, 2012).

## Analysis and Results

### Confirmatory Factor Analysis

Given our relatively small sample size, confirmatory factor analysis (CFA) was also carried out to support the discriminant validity of the four-factor model in AMOS 24.0. We evaluated model fit by using the various indices adopted by Li et al. (2010), including comparative fit index (CFI), non-normed fit index (NNFI), and root mean square error of approximation (RMSEA). To conclude that a model fits the data well, CFI and NNFI were suggested above the level of 0.90 (Chi and Qu, 2008), and the RMSEA value should below the acceptable level of 0.08 (Browne and Cudeck, 1992). The hypothesized measurement model was tested, and the indicators were loaded on each hypothesized latent construct (i.e., ESE, JS, IB, and ZY thinking). Results have proven that the hypothesized four-factor model (M0) displayed good fit with the data (χ^2^ = 697.376, *df* = 371, CFI = 0.925, NNFI = 0.912, RMSEA = 0.059). We also tested five alternative models to examine whether a more parsimonious model achieved an equivalent fit (Ng and Feldman, 2012). In comparison, alternative models all fit significantly worse than the four-factor model, and these indices fell short of the recommended standards (Table 2). Thus, it was concluded that the four factors were sufficiently distinct.

**TABLE 2:** Confirmatory factor analysis by comparing alternative measurement models.

Model	Description	*χ* ^2^	*df*	CFI	NNFI	RMSEA	△*χ* ^2^
M0	Hypothesized four-factor model: ESE, JS, IB, ZY	697.376	371	0.925	0.912	0.059	
M1	Three-factor model: JS and IB were combined into one factor	1,137.213	374	0.824	0.795	0.090	439.837**
M2	Three-factor model: IB and ZY were combined into one factor	1,232.142	374	0.802	0.769	0.096	534.766**
M3	Three-factor model: JS and ZY were combined into one factor	1,433.956	374	0.755	0.715	0.106	736.580**
M4	Two-factor model: JS, IB, and ZY were combined into one factor	1,864.734	376	0.656	0.602	0.126	1167.358**
M5	One-factor model: ESE, JS, IB, and ZY were combined into one factor	2,146.995	377	0.591	0.528	0.137	1449.619**

N = 249; CFI, comparative fit index; NNFI, non-normed fit index; RMSEA, root mean square error of approximation; ESE, entrepreneurial self-efficacy; JS, job satisfaction; IB, innovation behavior; ZY, Zhongyong thinking.**p < 0.01, two-tailed.

### Common Method Bias Control

Since all measurement scales were self-reported, there would be a potential for common method variance (CMV) caused by multiple reasons such as social desirability and consistency motif (Williams et al., 2003). In this study, the principles of confidentiality and voluntariness have been strictly followed to control the bias in research design as a procedural remedy. In addition, we performed statistical analyses followed by Liang et al. (2007) to assess the severity of CMV. First of all, Harman single factor analysis was conducted as a detection method (Carlson and Kacmar, 2000). If there is more than one factor extracted and the variance contribution rate of the first factor is no higher than 40%, it is generally considered that the deviation of the common method can be neglected (Podsakoff et al., 2003). The Harman single factor test has shown that the four factors of principal component analysis explained 63.40% of the total variance, among which factor one explained 39.11%, indicating that CMV is not a likely contaminant of our results.

Secondly, the inclusion and specification of a latent CMV factor is adopted to further detect the influence of CMV (Podsakoff et al., 2003; Williams et al., 2003). We included a latent CMV factor in the M0 model and observed the changes in model fit indices and each indicator’s variances substantively explained by the principal construct and by the method. In order to demonstrate that the results are not influenced by CMV, the addition of a CMV factor must not significantly improve the fit over the four-factor model (M0) (Ng and Feldman, 2012). Results showed that the CFA model with a CMV factor has an acceptable fit (χ^2^ = 566.920, *df* = 342, CFI = 0.948, NNFI = 0.934, RMSEA = 0.051). Though the overall chi-square statistics are significant, the gain in fit achieved by Model MCMV compared to M0 is relatively small (△CFI = 0.023, △NNFI = 0.022, △RMSEA = 0.008) (Facteau et al., 1995). In addition to relying upon the overall fit indices to assess CMV, this study calculated the average squared loadings of principal constructs and of the method factor loadings, which were interpreted as indicator variance caused by principal constructs and by method separately (Williams et al., 2003; Liang et al., 2007). Results from these analyses demonstrated that the percent of indicator variance caused by principal constructs is 42.75% in comparison with the average method-based variance of 15.70%, which is less than the amount of method variance (25%) observed by Williams et al. (1989). Therefore, CMV has not greatly affected this study.

### Reliability and Validity of the Scales

The reliability of the scales has been tested by using Cronbach’s α and composite reliability (CR) prior to the verification of the proposed conceptual model. Cronbach’s alpha was used to estimate the internal consistency reliability of each construct (Cheung et al., 2014). As shown in Table A1, Cronbach’s α values of SES, JS, IB, and ZY in this study were 0.840, 0.882, 0.872, and 0.896, respectively, all surpassing the threshold value of 0.70 (Kline, 1998). The values of CR were 0.809, 0.777, 0.792, and 0.801, respectively, meeting an acceptable level of construct reliability at 0.70 (Nunnally and Bernstein, 1994).

The convergent validity and the discriminant validity of the scales have been evaluated by calculating the average variance extracted (AVE) recommended by Fornell and Larcker (1981). Results showed that the factor loadings were all significant, and the load coefficient of each item ranged from 0.611 to 0.842. The AVEs ranged from 0.737 to 0.782, and all exceeded 0.50, thus confirming convergent validity (Fornell and Larcker, 1981). Meanwhile, each square correlation coefficient between factors was less than the AVE value for any two constructs, implying a satisfying discriminant validity (Cheung et al., 2014). Taken together, the reliability and the validity of the scales are sufficient for the next data analysis.

### Descriptive Statistics and Correlations

AMOS 24.0 and SPSS 22.0 statistical software were used to conduct data analysis in this study, with the results of descriptive statistics and correlation analysis of variables shown in Table 3. ESE was found to have a significant correlation with IB, and the correlation coefficient was 0.51 (*p* < 0.01). Meanwhile, ESE was also significantly and positively associated with JS (*r* = 0.42, *p* < 0.01), which also correlated greatly with IB (*r* = 0.56, *p* < 0.01). These results have provided preliminary support for subsequent hypothesis testing. In addition, experience in the start-up was largely correlated with gender, age, and education background, with gender (*r* = -0.13, *p* < 0.05) and education background (*r* = -0.29, *p* < 0.01) being negatively correlated and age being positively correlated (*r* = 0.21, *p* < 0.01).

**TABLE 3:** Descriptive statistics and correlations among the variables.

Variables	Mean	*SD*	1	2	3	4	5	6	7
1 Gender	1.44	0.50							
2 Age	2.41	1.29	–0.10						
3 Education background	3.10	0.72	0.04	−0.37**					
4 Experience in the start-up	2.20	0.84	−0.13*	0.21**	−0.29**				
5 Entrepreneurial self-efficacy	3.83	0.58	–0.11	0.13*	–0.03	0.10			
6 Job satisfaction	3.47	0.70	–0.02	0.06	0.05	–0.08	0.42**		
7 Innovation behavior	3.87	0.60	–0.04	0.18**	–0.11	0.07	0.51**	0.56**	
8 Zhongyong thinking	3.98	0.42	–0.02	0.09	–0.08	0.08	0.46**	0.43**	0.51**

N = 249. *Correlation is significant at the level of 0.05 (two-tailed). **Correlation is significant at the level of 0.01 (two-tailed). Gender: male = 1, female = 2. Age: ≤ 25 = 1, 26–30 = 2, 31–35 = 3, 36–40 = 4, ≥ 41 = 5. Education background: high school and below = 1; diploma = 2; bachelor = 3; master and above = 4. Experience in the start-up: ≤ 1 year = 1, 1–5 years = 2, ≥ 5 years = 3.

### Hypothesis Test

This study has applied hierarchical regression analysis to test the research hypothesis with SPSS 24 software, by firstly verifying whether ESE would positively affect IB when IB was set as the dependent variable. With control of gender, age, education background, and experience in the start-up (Model 1 of Table 4), ESE continued to be added to Model 2. The results demonstrated that H1 was supported with a reported significant association between ESE and IB in Model 2 (β = 0.51, *P* < 0.01).

**TABLE 4:** Regression analysis of hypotheses.

Variables	Innovation behavior				Job satisfaction			
	Model 1	Model 2	Model 3	Model 4	Model 5	Model 6	Model 7	Model 8
1 Gender	–0.03	0.02	0.02	–0.02	–0.04	0.02	0.00	0.00
2 Age	0.11*	0.08*	0.04	0.05	0.14**	0.11*	0.11*	0.11*
3 Education background	–0.04	–0.05	–0.08	–0.08	0.07	0.06	0.08	0.11
4 Experience in the start-up	–0.08	–0.08	–0.01	0.02	−0.19**	−0.19**	−0.20**	−0.19**
5 Entrepreneurial self-efficacy		0.51**	0.33**			0.50**	0.33**	0.31**
6 Zhongyong thinking			0.36**	0.48**			0.53**	0.47**
7 Entrepreneurial self-efficacy × Zhongyong thinking								0.33*
R^2^	0.04	0.28	0.42	0.34	0.04	0.21	0.28	0.30
Adjusted R^2^	0.03	0.27	0.41	0.33	0.02	0.19	0.27	0.28
F	2.68	19.22	29.62	25.03	2.36	12.67	16.00	14.80

N = 249. *Correlation is significant at the 0.05 level (two-tailed). ^∗∗^Correlation is significant at the level of 0.01 (two-tailed).

Secondly, JS was also set as a dependent variable to check whether it was positively affected by ESE. On the basis of controlling variables, Model 6 has suggested that ESE has a significantly positive effect on JS (β = 0.50, *P* < 0.01), thus to support H2.

Thirdly, the mediating effect of JS between ESE and IB has been tested by judging whether the following three conditions were met. That is, (1) ESE significantly correlates with IB; (2) ESE significantly relates to JS; and (3) when JS is included in the relation between ESE and IB, it is a complete mediation if the relation between ESE and IB is not significant while that between JS and IB is. Otherwise, ESE plays a partial mediating role, if there still exists the correlation between ESE and IB; however, the correlation coefficient decreases. This study has verified the significant effect of ESE on IB and JS respectively in Model 2 and Model 6. Model 3 has revealed that both ESE and JS influence IB to a great extent (β = 0.33, *P* < 0.01; β = 0.36, *P* < 0.01), and the coefficient correlation is less than 0.51 (Model 2) when JS is not included. Consequently, JS plays a partial mediating role in the correlation between ESE and IB, thus supporting H3.

**FIGURE 2:**
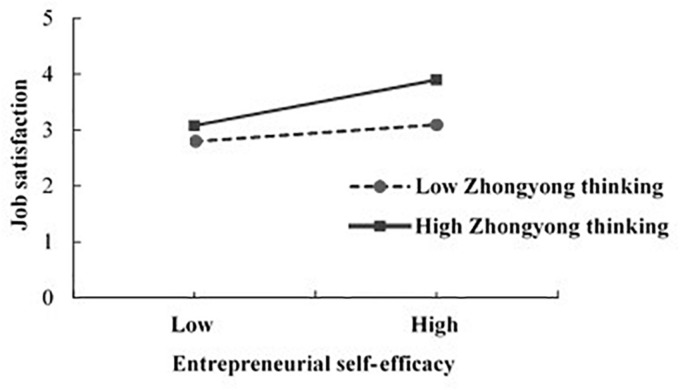
Moderating role of Zhongyong thinking in the relationship between entrepreneurship self-efficacy and job satisfaction.

We finally conducted regression analysis to test the hypothesis that the association between ESE and IB would be strengthened by ZY thinking. This study followed the moderated regression procedures recommended by Aiken and West (1991). Independent variables and ZY thinking were mean-centered ahead of analysis to reduce potential multi-collinearity problems. Meanwhile, we constructed the interaction of ESE and ZY thinking (ESE × ZY) and thus suggested the existence of the moderating effect when the coefficient of interaction was significant (Aiken and West, 1991). As shown in Models 5–8 in Table 4, we entered the control variables in Model 5, ESE in Model 6, ZY thinking in Model 7, and the interaction in Model 8. In addition to the core hypothesis of the relationship between ESE and JS, Model 7 showed that ZY was also a significant and independent predictor of JS (β = 0.53, *P* < 0.01). As predicted in Hypothesis 4, Model 8 identified that the interaction coefficient is significant (β = 0.33, *P* < 0.05), suggesting that ZY acts as a moderating role between ESE and JS. Simple slope tests were also conducted to further verify the interpretation of the interaction (Aiken and West, 1991). Figure 2 was plotted for the relationship between ESE and JS at one standard deviation above and below the mean of ZY. As expected, for individuals with a high sense of ZY (one standard deviation above the mean, 4.40–5), ESE significantly predicted a higher level of JS (*t* = 4.77, *P* < 0.01). On the contrary, the positive relationship between ESE and JS is weaker and reduced to non-significance (*t* = 0.70, ns) for entrepreneurs who exhibit less ZY tendency (one standard deviation below the mean, 1–3.56). These results indicate that the relationship between ESE and JS is positively moderated by ZY, and entrepreneurs with a higher sense of ESE are more likely to display JS when they also have a higher tendency of ZY thinking. In this sense, H4 is supported.

## Conclusion

IB is often regarded as an essential factor of the competitive advantage and potential of new enterprises. Therefore, it has become important for entrepreneurs to effectively stimulate and develop IB and then ultimately achieve self-realization. Many entrepreneurs with much entrepreneurial enthusiasm choose to quit midway, which makes scholars and entrepreneurs not only pay attention to whether ESE positively impacts IB but also be interested in how to effectively enforce the role of ESE. Despite the positive impact brought by ESE on a range of activities related to entrepreneurship (Dempsey and Jennings, 2014), we still lack an understanding of the cognitive process from ESE to IB, as well as relevant empirical studies. Hence, the Goal Self-Concordance Theory was adopted as a cognitive lens to study the relationship between ESE and IB as well as the mediating effect of JS and the moderating role of ZY thinking. Through a questionnaire survey, this study conducted empirical research based on 249 responses of start-up founders and top managers in China.

This study has reached the first conclusion that ESE has a positive effect on IB. The entrepreneurial process is often described as a goal pursuit focused on promotion (Brockner et al., 2004). Nevertheless, there is still little research on how to improve IB of entrepreneurs from the perspective of psychological factors in the field of entrepreneurship. Similar to the research of Chen and Zhou (2017), this study has generated evidence that ESE has a positive effect on promoting IB of entrepreneurs.

Secondly, this study has also proposed that JS is a considerable mediator between ESE and IB. As an important psychological quality of entrepreneurs, ESE affects not only the confidence and expectation of the innovation goal but also the attitude and behavior of entrepreneurs. Based on the agreement that generalized self-efficacy has a powerful effect on JS in existing studies (Jean and Mathieu, 2015), this study provides more evidence in view of China. Furthermore, empirical results have found that JS can partially mediate the relationship between ESE and IB. Compared with ordinary employees, entrepreneurs with higher self-efficacy also experience higher JS, indicating a better mental state of less pressure and emotional exhaustion, which leads to promoted IB. In other words, entrepreneurs with higher ESE, a psychological quality, gain psychological empowerment and positive feedback through higher JS, encouraging them to put continuous efforts on innovation.

The last conclusion is that ZY thinking has a meaningful moderating effect on the relationship between ESE and JS. Considering the characteristics of the entrepreneur group, analysis has also been conducted on the moderating effect of ZY in enabling entrepreneurs with higher ESE to obtain JS. The higher the ZY thinking of entrepreneurs, the stronger the effect of ESE on JS. In addition, the data also suggest a direct positive relation between ZY thinking and JS. These support the important role of ZY in mitigating damages caused by stress at work (Chou et al., 2014). As a cognitive strategy for entrepreneurs to relieve psychological pressure and emotional exhaustion, ZY helps to keep individuals in a more positive cognitive status, which allows them to realize higher JS during hard times.

## Discussion

This study has contributed theoretically by taking a close look at the process of ESE influencing IB. First, it is one of the few empirical studies to examine the effect of ESE on IB so far. From the perspective of an entrepreneur’s characteristics, we developed how the IB of entrepreneurs is affected by ESE and emphasized the significance of entrepreneurs’ psychological quality based on the empirical evidence from China. Our findings are consistent with the view that ESE has beneficial impacts on the activities related to entrepreneurship (Hmieleski and Corbett, 2008; Barz et al., 2015), indicating that ESE of entrepreneurs is one of the driving forces for their IB (Ahlin et al., 2013).

From the perspective of the cognitive process of ESE influencing behaviors, our study also contributes to both ESE and IB literatures by demonstrating the mediating effect of JS. We indicated that ESE can effectively influence IB through JS, suggesting that one effective process may be a positive attitude or feeling, which can further broaden the understanding of how ESE functions (Luthans and Ibrayeva, 2006). Although limited attention has been paid to JS of entrepreneurs in existing studies, JS is an outcome after evaluating different elements in the working environment, which for entrepreneurs are far more complex and riskier, compared with ordinary employees (Bhoganadam et al., 2016). Thus, this paper focuses more on the psychological experience and JS of entrepreneurs. Previous research on JS has shown that employees who are more satisfied and happier also have better job performance and IB (Bond and Bunce, 2003; Gallivan, 2003). Similar to the findings in the field of organizational behavior, our study shows that HS also exerts a positive effect on IB of entrepreneurs, increasing the general cognition that positive attitudes or feelings can promote beneficial behavior (Griffin et al., 2001; Lee and Chang, 2008; Li et al., 2010).

Moreover, this paper adds to the understanding of ZY thinking with a Chinese indigenous research perspective by examining its moderating role in the relationship between ESE and JS. Prior studies have shown cultural differences in ESE level, and the realization of entrepreneurial goals is also a process of internalizing national culture (Mcgee et al., 2009). To answer the research call of Sheldon et al. (2004) on the applicability of the Goal Self-Concordance Theory in different cultures, this paper enriches the mechanism research and application of the theory in the Chinese context by expounding on the connotation and regulating function of ZY thinking. This study provides a cognitive way to better understand the thinking way of many Asian entrepreneurs and their innovation behaviors, as cultural factors have a deep-rooted impact on individual cognition and behavior. As a characteristic of most Chinese thinking ways (Pierce and Aguinis, 2013), ZY thinking positively moderates the relationship between ESE and JS, which extends cultural boundary studies and enriches relevant research on dealing with entrepreneurship problems with ZY thinking. Different from most studies on ZY thinking conducted with employees and undergraduates (Yao et al., 2010; Chou et al., 2014; Qu et al., 2018), this study verifies the cognitive regulatory function of ZY thinking with entrepreneurs. Research on ZY thinking and other Chinese indigenous constructs, such as Guanxi and Yin-Yang (Lobo et al., 2013; Jing and Van de Ven, 2014), is beneficial to the interpretation of traditional Chinese Confucian culture (Pan and Sun, 2017). Compared with such concepts as collectivism and power distance which are put forward by westerners for cultural research (Tiessen, 1997; Vegt et al., 2005), this study can improve our understanding of how easterners speak out (Qu et al., 2018).

This study is also relevant to practitioners involved in entrepreneurship and makes some implications in practice. This paper firstly suggests how people with entrepreneurial ambitions can effectively improve their IB. Specifically speaking, they should build up their confidence in completing tasks related to entrepreneurship by continuous learning, practicing, and reflecting, which would further improve their psychological quality and form a virtuous circle of ESE influencing IB (Chen et al., 1998). Secondly, the ability of entrepreneurs to enhance psychological quality and properly deal with entrepreneurial stress is crucial to a successful entrepreneur. The key of turning existing psychological quality to competitive advantages for entrepreneurs lies in their positive psychological cognition, and an effective way to convert ESE to UB is to concentrate on enhancing JS. Research findings further suggest that the feeling of JS can be improved by cultivating and promoting ZY thinking. From the perspective of culture studies, this paper emphasizes the important role of ZY thinking in interpreting the cognition and behavior of Chinese entrepreneurial groups and even most Asian groups and provides an academic foundation for cross-cultural communication and cooperation. In addition, the supportive role played by other groups is also indispensable since it is a challenging process for entrepreneurs to relieve pressure by themselves (Powell and Eddleston, 2013). Families and friends should provide more trust and support to entrepreneurs to reduce their psychological pressure and emotional exhaustion, since a balance between family, friendship, and entrepreneurship is also crucial to carry out entrepreneurial activities successfully. As the main institution and department of entrepreneurship education, it is necessary for universities and the government to provide business guidance and resources but also emphasize the psychological quality and emotion management of entrepreneurs. More importantly, this paper also provides a reference for a wider range of entrepreneurs and industries in other cultures. Especially in the rapidly changing entrepreneurial environment, ZY thinking is closely related to adaptive capacity and can be considered as a cognitive strategy to effectively cope with a changing and uncertain environment (Pan and Sun, 2017). ZY thinking can be conducive to individuals in coping with entrepreneurial pressure, integrating resources, and implementing suitable methods (Zhou et al., 2019). Such awareness may help individuals focus more on the positive than the negative side and be more likely to exchange information, thus inspiring and sustaining innovation behaviors.

## Limitations and Future Research

Although this paper has shed light on the understanding of how ESE effectively affects IB to a certain extent, there still are several limitations in need of attention. This study has introduced a mechanism linking ESE and IB according to the Goal Self-Consistency Theory, and the possibility of the existence of other potential mechanisms cannot be ruled out. Hopefully, there can be more mediation mechanisms explored in the research of the relationship between ESE and behaviors in relevance to entrepreneurship, for which more path analysis and multi-level analysis are required. More variables that give positive psychological feedback to entrepreneurs can be further studied, such as trust and family support (Smith and Lohrke, 2008; Powell and Eddleston, 2013).

Chinese entrepreneurs are an active innovation community in the global environment. All the participants involved in this study are from China, and research on the cultural boundary condition was conducted via adopting ZY thinking as a moderating variable. Thus, the applicability of the model with cultural characteristics proposed in this paper needs to be further verified. Opportunities for future research have been inspired from a comparative study of different cultures between east and western countries and the feasibility of the cognitive strategy of ZY thinking in other cultural contexts.

Apart from that, the data are cross-sectional and thus do not establish causality in relationships. Limited focus was put on the time slot when filling out the questionnaire, but the process by which ESE affects IB is time-based. ESE of entrepreneurs may be affected by other factors over time, such as entrepreneurial education and entrepreneurship experience (Piperopoulos and Dimov, 2014; Kasouf et al., 2015); thus, future research could conduct longitudinal tracking studies to obtain samples and data from different time slots.

## Data Availability Statement

The datasets generated for this study are available on request to the corresponding author.

## Ethics Statement

Ethical review and approval was not required for the study on human participants in accordance with the local legislation and institutional requirements. The patients/participants provided their written informed consent to participate in this study.

## Author Contributions

JW participated in the design, data collection, drafting of the early version, and revising of the manuscript. YC participated in the data collection and analysis, drafting of the early version, and revising of the manuscript. YZ and JZ participated in the design and revising of the manuscript.

## Conflict of Interest

The authors declare that the research was conducted in the absence of any commercial or financial relationships that could be construed as a potential conflict of interest.

## Appendix

**TABLE A1:** Measurement for variables.

Reflective construction	Standardized loadings (λ)*
Entrepreneurial self-efficacy (AVE = 0.782; *R* = 0.809).	
I know about start-ups and the activities during entrepreneurship.	0.773
I am able to choose suitable employees for my business.	0.839
I am able to come up with new ideas to solve problems in entrepreneurship.	0.793
I have confidence in my ability to solve problems in my business.	0.760
Job satisfaction (AVE = 0.739; CR = 0.777).	
I am satisfied with the opportunities which exist in this organization for advancement.	0.783
I am satisfied with the relations with others in the organization with whom I work.	0.786
I am satisfied with the person who supervises me.	0.842
I am satisfied with the nature of the work I perform.	0.835
I am satisfied with the payment I receive for my job.	0.815
Considering everything, I am satisfied with my current job situation.	0.812
Innovation behavior (AVE = 0.749; CR = 0.792).	
I always seek to apply new processes, techniques, and methods.	0.829
I often come up with creative ideas.	0.808
I often communicate with others and present my new ideas.	0.757
In order to implement new ideas, I can find ways to get the resources I need.	0.751
In order to realize new ideas, I can make suitable plans.	0.774
Generally speaking, I am an innovative person.	0.773
Zhongyong thinking (AVE = 0.737; CR = 0.801).	
When discussing, I will consider conflicting opinions at the same time.	0.724
I am used to thinking about the same thing from different perspectives.	0.611
I will listen to all opinions before I express them.	0.738
When I make a decision, I will consider various possible conditions.	0.671
I often try to find acceptable opinions in a situation of disagreement.	0.733
I often try to find a balance between my own opinions and those of others.	0.714
I will adjust my original ideas after considering the opinions of others.	0.680
I expect to reach a consensus during the discussion.	0.699
I try to incorporate my own opinions into the thoughts of others.	0.676
I usually express conflicting opinions in a tactful way.	0.662
I will try to reconcile the minority to accept the majority in a harmonious way.	0.694
I usually consider the harmony of the organizational climate before making a decision.	0.731
I usually adjust my behavior for overall harmony.	0.706

*AVE, average variance extracted, CR = composite reliability. *All standardized loadings are significant (p < 0.001).*
